# Enamel Microcracks Induced by Simulated Occlusal Wear in Mature, Immature, and Deciduous Teeth

**DOI:** 10.1155/2018/5658393

**Published:** 2018-04-16

**Authors:** Manhal Ijbara, Kanae Wada, Makoto J. Tabata, Junichiro Wada, Go Inoue, Michiyo Miyashin

**Affiliations:** ^1^Pediatric Dentistry Department, Division of Oral Restitution, Track of Medical and Dental Sciences, Graduate School of Medical and Dental Sciences, Tokyo Medical and Dental University, Tokyo, Japan; ^2^Biostructrual Science Section, Bio-Matrix Department, Track of Medical and Dental Sciences, Graduate School of Medical and Dental Sciences, Tokyo Medical and Dental University, Tokyo, Japan; ^3^Removable Partial Denture Department, Track of Medical and Dental Sciences, Graduate School of Medical and Dental Sciences, Tokyo Medical and Dental University, Tokyo, Japan; ^4^Cariology Department, Track of Medical and Dental Sciences, Graduate School of Medical and Dental Sciences, Tokyo Medical and Dental University, Tokyo, Japan

## Abstract

Enamel wear, which is inevitable due to the process of mastication, is a process in which the microcracking of enamel occurs due to the surface contacting very small hard particles. When these particles slide on enamel, a combined process of microcutting and microcracking in the surface and subsurface of the enamel takes place. The aim of this study was to detect microscopic differences in the microcrack behavior by subjecting enamel specimens derived from different age groups (immature open-apex premolars, mature closed-apex premolars, and deciduous molars) to cycles of simulated impact and sliding wear testing under controlled conditions. Our findings indicated that the characteristics of the microcracks, including the length, depth, count, orientation, and relation to microstructures differed among the study groups. The differences between the surface and subsurface microcrack characteristics were most notable in the enamel of deciduous molars followed by immature premolars and mature premolars whereby deciduous enamel suffered numerous, extensive, and branched microcracks. Within the limitations of this study, it was concluded that enamel surface and subsurface microcracks characteristics are dependent on the posteruptive age with deciduous enamel being the least resistant to wear based on the microcrack behavior as compared to permanent enamel.

## 1. Introduction

Enamel is the hardest mineralized tissue in the human tooth and the human body, with a mineral content of 87% by volume and 96% by weight. With this high mineral content, enamel is expected to exhibit limited resistance to wear and fatigue crack growth [1]. In fact, the observation of cracks and/or occlusal wear facets in the clinical or laboratory examination of teeth is not uncommon. Surprisingly, these features seldom cause tooth fracture as enamel has excellent mechanical antiwear properties [2]. A review [3] explained the classification and mechanisms of enamel's wear as a process of surface microcracking. Microcracks occur when enamel surfaces contact very small hard particles. While the particles slide on the surface, a combined process of microcutting, cutting of enamel crystals at the level of rods and interrods microstructures, and microcracking of both the surface and the subsurface takes place. This cumulative process of plasticity-induced microcrack generation and coalescence is referred to as microcracking mode and/or severe wear [4].

The scientific literature on enamel wear characteristics and mechanical behavior is scant [5]. Understanding those characteristics is essential for guiding both scientists and dentists in selecting appropriate materials for dental restoration which have better friction matching properties [2]. To match the physical properties of enamel is challenging. In addition to having a complex hierarchical structure with macro- and microfeatures [6], the chemical composition of the outer layer of enamel changes intraorally. This chemical composition change, known as “posteruptive maturation,” influences the chemical and physical properties of deciduous and permanent enamel [7, 8]. Wear resistance and microcrack behavior are essential physical properties. Accordingly, we hypothesized that characteristics of the wear induced microcracks in enamel subjected to the same wear stimulating conditions will differ according to the posteruptive age.

In this study, we conducted an enamel to enamel, three-bodied simulated wear test with controlled conditions of temperature, frequency, force of impact, number of cycles, and specimens' size. The enamel specimens were obtained from deciduous molars, immature premolars (teeth have erupted in the mouth but the process of root morphogenesis is incomplete, a process which takes up to three years after eruption; during this stage, the apex of the root is considered to be open), and mature (fully formed, closed-apex) premolars. The aim of this study was to detect microscopic differences in the behavior of the wear induced microcracks between the study's specimens.

## 2. Materials and Methods

This study protocol was approved by the ethics committee of Tokyo Medical and Dental University (Institutional Research Board approval number: 02016-069). The authors of this paper declare no conflicts of interest in association with the present study (Form number 965-6).

### 2.1. Specimens' Preparation

This methodology was adapted from previously published studies [9, 10]. Forty-two sound, caries-free, wear facets-free immature premolars with open apices from patients of 9–15 years of age, forty mature/fully formed premolars from patients of 20–30 years of age, and twenty deciduous molars were used in this study. The teeth were extracted for reasons other than this study and stored in distilled water with a few Thymol crystals and were checked microscopically to exclude any wear facets or microcracks caused by the extraction forceps. Premolar teeth were selected with comparable buccolingual dimensions to minimize size discrepancies. The roots were cut using a high-speed diamond fissure bur with copious cooling water. The pulp cavities of the crowns were cleaned, etched, and filled with flowable resin composite (Metafill Flo, Sun Medical Co., Ltd., Tokyo, Japan). Half of the crowns in each group (immature [IM], *n* = 21; mature [M], *n* = 20; deciduous [D], *n* = 10) were embedded in clear self-curing resin (Unifast III Clear, GC Corporation, Tokyo, Japan) in the center of acrylic rings with the bucco-occlusal cuspal areas against a glass slab surface. After the resin cured, the lower surface of the ring was polished using abrasive papers mesh order of 600, 800, 1000, 1200, and 1500, with copious irrigation. This created an exposed enamel window of at least 2 mm^2^ (minimum, 2 mm × 1 mm) through the cured acrylic resin which was rechecked microscopically to ensure a glossy finished surface with absence of microcracks. These specimens were considered the study group and stored in distilled water in order to be placed in the lower compartment of a wear simulating machine. Another set of specimens was prepared from the buccal halves of the remaining crowns. Each half was bonded to a metal stylus with Super Bond C&B (Sun Medical Co., Ltd., Tokyo, Japan). Using a custom-made fine-grit cup-shaped diamond bur (Hinatawada Seimitsu Mfg. Co., Ltd., Tokyo, Japan) with copious cooling water, the bucco-occlusal surfaces of the cusps were ground to a hemisphere of 5 mm in diameter. These specimens were stored in distilled water to be used on the upper handles of the wear test machine.

### 2.2. Impact Sliding Wear Testing (ISWT)

The metal styli of the upper specimens were placed on the force application handles of an impact sliding wear testing machine (ISWT K655-07, Tokyo Giken, Japan). The acrylic rings of the lower specimens were fixed at the bottom of the water-filled tank of the ISWT machine. The machine was set to direct the upper enamel specimens from initial contact position with the enamel windows of the lower specimens to drop from a height of 1 mm with a force of 30 N, twice. After impacting, the upper specimens will slide for 1 mm horizontally (mesiodistally and back) twice, before rising up to the beginning of the cycle. A total of 20000 cycles were performed with a frequency of 0.23 Hz. The entire impact sliding test took place below the water level in the tank of the machine. The water temperature and level were checked for heating and evaporation at 37°C. The lower specimens were covered by a slurry of polymethyl methacrylate (PMMA) powder (Techpolymer MBX-50, Sekisui Plastics Co., Ltd., Tokyo, Japan) mixed with tap water at a 1 : 1 weight ratio to mimic a food bolus during the wear test. Every 5000 cycles, the specimens' positions were checked and the slurry was washed off and renewed.

### 2.3. Specimens' Imaging

After ISWT, all specimens were washed in distilled water for 20 seconds to remove any remnant slurry and dried with a gentle air spray. No polishing was performed as it might cause further damage to the existing wear features or produce new microcracks. A scanning laser confocal microscope (SCLM; 1 LM 15 W, with Nikon lenses, Lasertec Corp, Tokyo, Japan) was first used to take images of the enamel windows. A ×5 Nikon lens was used to capture the images of the microcracks, with a minimum measurement scale of 0.302 *μ*m. The other lenses (×10, ×20, and ×50) were used to capture images of the microcracks pathway throughout the enamel windows. Specimens were then examined utilizing a 3D colored laser microscope and profilometer (VK 250 series, Keyence Company, Tokyo, Japan) using magnification lenses in the order of ×5, ×10, and ×20. The microcracks lengths and depths as well as the morphology and dimensions of the wear zone were measured with the minimum scale accuracy on the *z*-axis set at 0.005 *μ*m. Three-dimensional images (3D images) of the specimens' surfaces were also constructed. To reveal the subsurface microcracks, specimens were examined using a fiber-optic stereomicroscope (Leica MZ10 F, LEICA Corp., Tokyo, Japan). Multiple images were taken with different illumination/transillumination directions for each specimen to ensure maximum contrast. Finally, the specimens were prepared and examined under a scanning electron microscope SEM (H-4500, Hitachi High-Technologies Corporation, Tokyo, Japan) at magnifications of ×5000, ×10000, and ×20000 in order to examine the relationship between the microcracks and the enamel microstructure.

### 2.4. Statistical Analysis

Count and length of surface and subsurface microcracks as well as depth, wear area, and enamel window area were analyzed (IBM SPSS Statistics for Windows, version 24, IBM Corp., Armonk, NY, USA). Kolmogorov–Smirnov test showed nonparametric distribution of the data. Kruskal Wallis test was used to compare between tested groups followed by Mann–Whitney *U* test for pairwise comparison with the significance level set at 0.05.

## 3. Results

### 3.1. Microcracks and the Wear Characteristics of the Specimens

**Figure 1:**
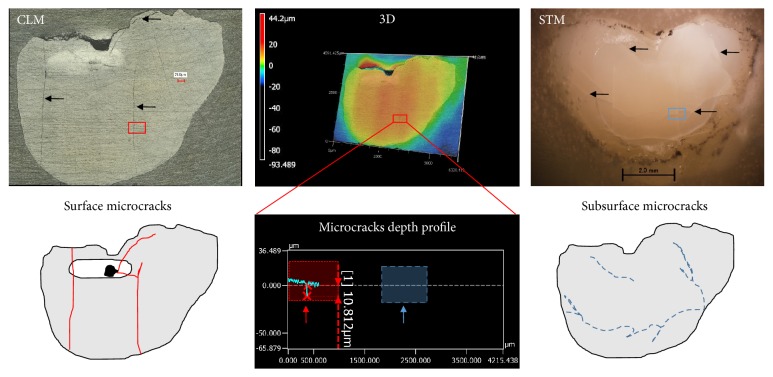
*Detection of subsurface microcracks with different imaging techniques*. A scanning microscope was used to detect and capture details and profile of the surface microcracks. Subsurfaces microcracks (those that did not reach the surface and were not detected by the scanning microscope as shown on the microcrack depth profile) were detected by a stereomicroscope with different directions of illumination/transillumination using fiber-optic lights. Images obtained by different imaging techniques were traced, measured, and analyzed. CLM: colored laser microscope. STM: stereomicroscope. 3D: three-dimensional construction of the surface and profile of one of the surface microcracks (the schematic presentations of the surface microcracks are in red, tracings of the subsurface microcrack are in blue, for explanatory purposes only; arrows indicate microcracks positions).

**Figure 2:**
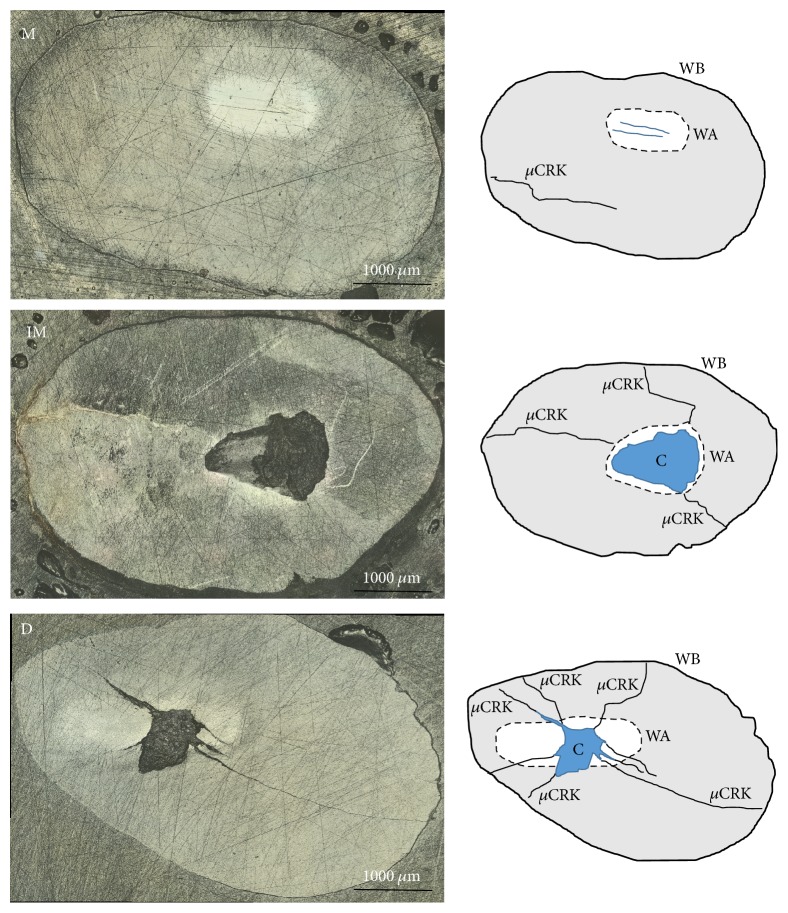
*The outcomes of ISWT on each group*. The schematic representation to the right of each group is a simplistic differentiation tool to compare the images of surface microcracks. M: mature enamel. IM: immature enamel. D: deciduous Enamel. WB: enamel window border. WA: wear area. C: crater, *μ*CRK: surface microcracks are shown with their corresponding schemes (scale bar = 1000 *μ*m).

The scanning laser microscope/profilometer had a wide field of imaging. This was useful for capturing the length and depth of the surface microcracks and the orientation toward the shape of the wear zone. In contrast, the scanning confocal microscope had a limited field of imaging but was efficient in capturing the finer details of the microcrack pathways and differentiating large scratch marks. The stereomicroscope was the only suitable microscope for revealing subsurface microcracks. Changing the direction of the light revealed the presence of multiple microcracks that did not reach the surface (Figure 1). Impact sliding wear had its effect on each group. Group M (80%) consistently showed a flat shiny oval wear zone without craters. Scratches or furrows were present in the wear zone with enamel rods apparent at low magnifications (×5). Microcracks were minimal, unbranched, and parallel to the direction of the sliding wear movement. Group IM (72%) tended to show a triangular wear zone that was mostly occupied by a crater. Multiple, branched microcracks were observed to radiate from the angles of the wear area; three specimens (14.2%) showed a network of microcracks to one side of the wear area. Group D (all specimens of the group) showed a flat wear zone but unlike mature group, crater or deep microcracks were present within the wear zone. The direction of those deep microcracks or craters was perpendicular to the direction of sliding. Group D suffered extensive microcracking on both surfaces and the subsurfaces (Figure 2).

### 3.2. Microcrack Counts, Total Lengths, and Depths

Table 1 shows the different results obtained from the microcracks analysis. Group M scored the lowest microcrack count and the shortest total microcrack lengths on both the surface and subsurface zones, significantly lower than those in Group D (*p* ≤ 0.001 for all parameters). The values of Group IM were intermediate between those of Groups D and M. Although the surface microcrack count and total length were significantly different from the values of Group M (*p* ≤ 0.001), the subsurface microcracks in Group IM were significantly different from Group D (*p *≤ 0.001). The differences in the depth of the surface microcracks were only significant near the wear zone in Group D (*p* = 0.005). The depth of the microcracks in Group D was 3-fold deeper than the other two groups.

## 4. Discussion

The present study investigated the susceptibility of enamel to wear according to the characteristics of induced microcracks. Enamel specimens with longer, deeper, and more numerous microcracks were considered more prone to wear. The results showed that each group presented with different patterns/measurements of microcracks, wear zones, and craters. This confirmed our hypothesis regarding the effect of the posteruptive age on enamel microcracks' behavior.

Microcracking is an essential component in the mechanism of enamel wear. Studying enamel microcracking is challenging since wear is a multifactorial, complex, unpredictable, and uncertain process [1, 11, 12]. Various factors, including impact forces [12], sliding forces [4], indentation forces [13], wet and dry conditions [14], and the presence of bolus [9], have been addressed in the literature in order to mimic/simulate the intraoral wear process. Fewer studies have been conducted to study the differences in the enamel properties obtained from different age groups, or the wear induced by enamel to enamel contact [1].

Wear versus fracture is a key issue in understanding the mechanical response of enamel [15]. In order to ensure the production of surface microcracks rather than total enamel fractures, the scale factor, that is, the magnitude of force and size of particle, should be taken into consideration. A small hard particle contacting the enamel surface with low force will cause surface removal (i.e., wear) by the processes of local deformation and associated microcracking. Plastic deformation and surface microfracture that are unidentified by the naked eye will accumulate over time until they become clinically visible [3]. This study used spherical slurry particles (size: 50 *μ*m) with forces that were considered low in comparison to masticatory forces, in order to prevent enamel fractures. The limited number of cycles prevented the penetration to the dentin in the deciduous specimens [10]. The microcutting effect of the slurry particles presented as fine scratching marks or furrows [16] in all groups.

In the present study, impact/sliding forces played a role in the formation of a deep crater in the wear zones of the immature and deciduous groups. High magnification images of the craters indicated rugged surfaces with severe damage to the microstructures; no identification of enamel rods or interrod was possible. On the other hand, in the mature group polished/shiny wear zones were observed more frequently than craters. Enamel microstructures were detected as being compressed with less damage. A previous study reported similar findings noting that enamel microstructure was compressed but preserved after sliding wear testing against a titanium alloy in adult teeth [12]. Those findings might be attributed to differences in the chemical composition of enamel and its effects on the mechanical properties [8, 17]. Enamel will increase in density, increase in crystallinity, and decrease in permeability over intraoral time [18]. This aging process of enamel in the oral cavity might explain the resistance of the mature group to crater formation, microcrack formation, and microcrack extension in comparison to the immature and deciduous groups.

In the present study, ISWT caused microcracking of the subsurface in all of the specimens. Similar findings suggested that subsurface microcracks coalesce in severe wear [4], contrary to another study [12] which indicated that no subsurface cracks were involved after wear testing due to the cushioning effect of water content. Since no sectioning was performed on the specimens after the ISWT (to prevent any further damage or uncontrolled microcracking) the origin of the subsurface microcracks was not clear. Other studies reported that subsurface cracks were related to radial cracks [13, 19]. Radial cracks initiate from the base of flexing brittle enamel and extend upward toward the surface [3, 11, 13, 19]. The deciduous group suffered more numerous and extensive subsurface microcracks in comparison to the other two groups. A study [17] reported that microcracks readily formed in the deciduous enamel subsurface at low indentation forces due to its poor fracture resistance in comparison to adult enamel.

**Figure 3:**
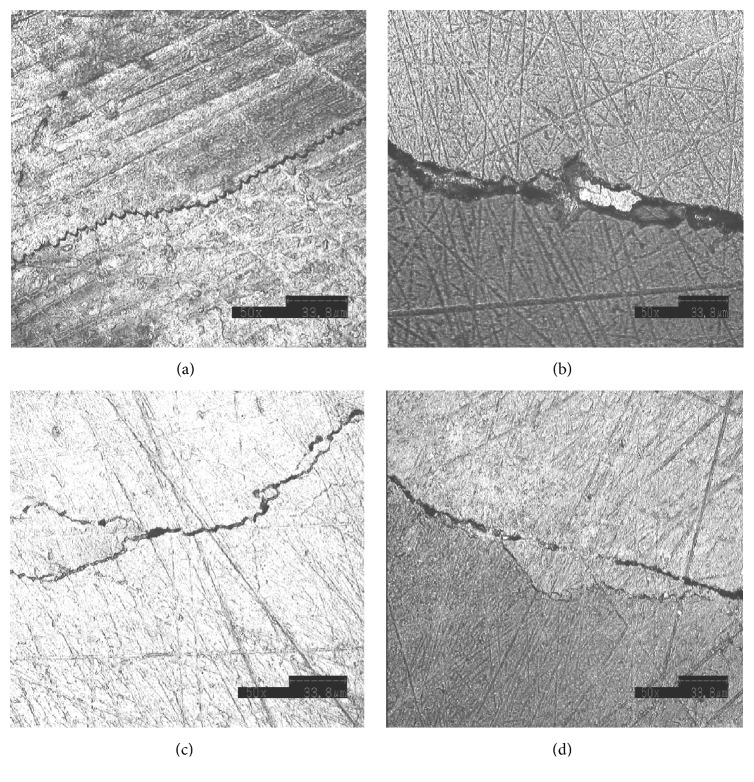
*The enamel mechanisms of defense along the microcrack pathways*. Different obstacles to microcracks were found in the enamel microstructure of all specimens. (a) Scalloping of the microcrack indicated that the crack followed the interrod spaces. (b) Bridging is defined as the incomplete separation of the enamel microstructure or ligaments. (c) The pathway of the microcrack was curved rather than straight. (d) Bifurcation of microcracks which divides the driving force between two cracks (rather than one) through the enamel microstructure.

The analysis of both SEM and SCLM images revealed the relationship between microcracks and the enamel microstructure at the micron and submicron levels. Microcracks showed a scalloping pathway through interrod spaces in all groups. The interrod area is filled with proteinous materials with reported roles in self-healing, cushioning effect, and lengthening cracks pathways as defense mechanisms to diminish cracks driving forces [19–23]. A comparison of the enamel of 18–30 years of age and that of enamel of ≥55 years of age [18] revealed that a reduction of this protein interrod matrix or its replacement with fluoroapatite crystals that occurred with age was associated with increased density, increased hardness, reduced permeability, and reduced fatigue toughness. However, in our study (due to the younger age groups) we found that the microcrack resistance of enamel increased with time. Aside from scalloping, microcracks faced curvatures, bridging, and bifurcations [1]; there was a tendency for the width of the gap between its edges to change within the same pathway in all groups (Figure 3). Two studies [21, 24] found that crack deflection and bifurcation were more prominent as anticrack defense mechanisms than the bridging mechanism in enamel of more than 50 years of age. Unfortunately this was not an objective in this study.

In the present study, microcracks obtained superficial positions along their pathway along the enamel surface. Two reports discussed the mineral changes that were detected beyond the surface of premolar teeth at depths of 25–60 *μ*m [25, 26]. The maximum depth of microcracks near the wear zone was within this depth range in all groups.

**Figure 4:**
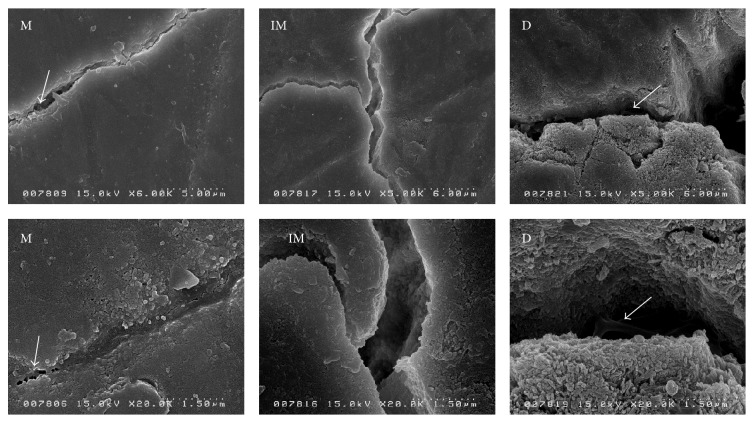
*The effect of microcracks on the enamel microstructures*. SEM images of specimens at different levels of magnification reveal the effect of microcracks on the enamel microstructure. The surface of the mature enamel showed shallow microcracks and minimal bifurcation, with minimal damage to the microstructure of the wall and some detachment of the enamel crystals from the wall edges. Immature enamel had deeper microcracks, moderate bifurcation, and moderate damage to the microstructures on the microcracks edges. Deciduous enamel suffered deep microcracking, severe bifurcation, and detachment of not only the crystals but also the enamel rods on the edges of microcracks. M: mature tooth specimen. IM: immature tooth specimen. D: deciduous tooth specimen (the scale is indicated at the bottom of each image; white arrows indicate enamel ligaments).

The SEM images of the microcrack edges showed differences among the study groups (Figure 4). Deciduous enamel crystals were larger, less arranged, and more detached from the microcrack edges with enamel rods separated in the walls of the microcracks. Immature and mature specimens showed enamel crystals that were ordered, packed, and compressed rather than detached, with the rods showing more stability in comparison to those in the microcrack walls. It was reported that deciduous enamel crystals were larger than permanent enamel crystals (185 nm versus 94 nm, resp.) [17], with less calcium/phosphate content and more carbonated apatite [27]. Deciduous enamel was more permeable, with abundant micropores, larger interrod areas, and rods that were smaller and less dense [6, 8]. These chemical, microstructural, and crystalline differences will reduce the toughness and hardness of deciduous enamel, reducing its resistance to microcracking. SEM imaging detected the presence of protein enamel ligaments bridging the edges of the microcracks in some deciduous specimens (Figure 4). These protein ligaments act as an intrinsic self-repair mechanism and a location for mineralization in the inner enamel [22]. In the mature group, these enamel ligaments were of a different “mineral” type. Mineral ligaments bridge microcracks at the crystalline level with enamel proteins acting as a natural adhesive between the crystals [23].

To the authors' knowledge, this is the first study that attempted to quantitatively and qualitatively evaluate microcracks induced by simulated occlusal wear among mature premolars, immature premolars, and deciduous molars, that is, different age groups. It might be also the first attempt to evaluate the wear of deciduous enamel's mechanically. This might be attributed to the limited volume of enamel, which hinders the desired size and geometry of any enamel specimen that can be obtained [28]. Although the mechanical and compositional differences between adult teeth and deciduous teeth have been discussed [6, 7, 17, 27], none compared the enamel of immature premolars to mature premolars. One study on third molars showed that enamel became less resistant to fracture with increasing age (17–25 years of age versus ≥55 years of age) [24]. Our findings indicate that enamel becomes more resistant against microcracking with time at younger age groups (from adolescence to 30 years). Primary enamel is generally considered softer and more wear prone in comparison to permanent enamel [8]. However, the comparison is difficult due to the variety of hardness testing techniques applied in previous studies and the anisotropic nature of enamel microstructures [29]. In Vickers microhardness tests, primary enamel surface hardness was lower when compared to mature enamel (≤2.9 GPa versus ≥3.4 GPA, resp.) [17]. Similar findings were reported previously as permanent enamel has had higher surface hardness with a range of 3 to 6 GPa [30]. One study [2] compared hardness of enamel surface in four different age groups (primary teeth, permanent teeth at young age, middle age, and old age). It was noted that primary enamel had lower hardness than permanent teeth. Moreover the hardness of young and middle aged permanent enamel was highest among the study groups.

Although this study had a merely extrinsic, nondestructive approach of its methodology, differences in the microcracks behavior in relation to the posteruptive age of enamel, mature, immature, and deciduous groups, were detected. Accordingly, differences in the intrinsic toughening mechanism of enamel, that is, the exact role of the enamel subsurface microstructures in wear and microcrack resistance, in relation to the posteruptive age are in need of further investigations. Our future research plans include a micromechanical approach in examining the microcracks characteristics in relation to posteruptive age; that is, tests as nanoindentation, microtoughness, and microfracture might be included.

## 5. Conclusions

Within the limitations of this study we concluded the following. Enamel surface and subsurface microcracks characteristics are dependent on the posteruptive age. Although deciduous enamel is least resistant to wear based on microcracks characteristics in comparison to permanent enamel, there are still significant differences in the wear resistance of the permanent enamel at different eruptive ages which require further investigations by a microstructural approach.

## Figures and Tables

**Table 1:** * The results obtained from the analysis of the microcrack parameters in each specimen*. The total length of microcracks, count/number of microcracks, and depth of microcracks in the surfaces and subsurfaces of the study groups.

Parameter		Groups			*p* value
		Mature	Immature	Deciduous	
Number of surface microcracks	Median	4	7	9	
	Range	5	10	7	
	Maximum	7	13	13	
	Minimum	2	3	6	
	Rank^†^	a	b	b	≤0.001^*∗*^

Total length of surface microcracks (mm)	Median	5.64	10.80	16.06	
	Range	10.44	15.89	17.25	
	Maximum	11.37	18.17	25.13	
	Minimum	0.93	2.28	7.88	
	Rank	a	b	b	≤0.001^*∗*^

Number of subsurface microcracks	Median	2	3	6	
	Range	7	6	5	
	Maximum	7	6	9	
	Minimum	0	0	4	
	Rank	a	a	b	≤0.001^*∗*^

Total length of subsurface microcracks (mm)	Median	2.69	3.77	12.15	
	Range	6.20	9.90	24.98	
	Maximum	6.20	9.90	29.94	
	Minimum	0.00	0.00	4.96	
	Rank	a	a	b	≤0.001^*∗*^

Depth of microcracks near wear area (*µ*m)^††^	Median	4.37	4.38	14.99	
	Range	17.04	25.68	24.43	
	Maximum	17.98	26.49	28.42	
	Minimum	0.94	0.81	3.99	
	Rank	a	a	b	0.005^*∗*^

Depth of microcracks far from wear area (*µ*m)	Median	2.34	2.76	2.88	
	Range	14.33	15.01	5.12	
	Maximum	14.85	15.25	7.62	
	Minimum	0.52	0.24	2.50	
	Rank	a	a	a	0.711 NS

*∗* indicates a statistically significant difference. ^†^The lowercase letters in the columns indicate statistically significant intergroup differences in the same characteristics in rows. ^††^The depth of surface microcracks shows the deepest microcrack measurement in relation to the position of the wear area.

## Supplementary Materials

Supplementary Figure * *1
* A schematic representation of the preparation of the upper and lower specimens*. A: the upper specimens were prepared by grinding the bucco-occlusal surfaces of the cusps and cutting the formed enamel hemispheres; they were then cemented on metallic styli. B: the lower specimens (study groups) were prepared by embedding crowns in acrylic rings and then molded in acrylic resin. C: actual images of the upper and lower specimens after polishing.* Footnote of Supplementary Figure  1*. The dotted line indicates the cutting direction; the red line indicates the final position of the crown.

Supplementary Figure * *2
* Impact sliding wear testing (ISWT)*. Positions of the upper and lower enamel specimens below the water level in an impact sliding wear machine chamber. The whitish slurry of PMMA powder was placed in between specimens. The cyclic impact and sliding movements of the upper specimen against the lower specimen in relation to the initial contact position are indicated by numbered arrows (each arrow represents 1 mm).

Supplementary Figure * *3
* Images of the enamel window of a deciduous enamel specimen under different microscopes*. A: a stereomicroscope view. B: a scanning laser microscope and profilometer. C: a scanning confocal laser microscope. D: three-dimensional images were constructed with a depth scale (note the different scale measurements of each microscopic image).

## References

[1] Arola D., Bajaj D., Ivancik J., Majd H., Zhang D. (2010). Fatigue of biomaterials: Hard tissues. *International Journal of Fatigue*.

[2] Zheng J., Zhou Z. R. (2006). Effect of age on the friction and wear behaviors of human teeth. *Tribology International*.

[3] Lucas P. W., Van Casteren A. (2015). The wear and tear of teeth. *Medical Principles and Practice*.

[4] Borrero-Lopez O., Pajares A., Constantino P. J., Lawn B. R. (2014). A model for predicting wear rates in tooth enamel. *Journal of the Mechanical Behavior of Biomedical Materials*.

[5] Gao S. S., An B. B., Yahyazadehfar M., Zhang D., Arola D. D. (2016). Contact fatigue of human enamel: Experiments, mechanisms and modeling. *Journal of the Mechanical Behavior of Biomedical Materials*.

[6] Zamudio-Ortega C. M., Contreras-Bulnes R., Scougall-Vilchis R. J., Morales-Luckie R. A., Olea-Mejía O. F., Rodríguez-Vilchis L. E. (2014). Morphological, chemical and structural characterisation of deciduous enamel: SEM, EDS, XRD, FTIR and XPS analysis. *European Journal of Paediatric Dentistry*.

[7] Sabel N. (2012). Enamel of primary teeth—morphological and chemical aspects. *Swedish Dental Journal Supplement*.

[8] Lynch R. J. (2013). The primary and mixed dentition, post-eruptive enamel maturation and dental caries: a review. *International Dental Journal*.

[9] Suzuki S. (2004). Simulated enamel wear during occlusal contact. *American Journal of Dentistry*.

[10] Wada K., Miyashin M., Nango N., Takagi Y. (2011). Wear of resin composites and primary enamel and their applicability to full crown restoration of primary molars. *American Journal of Dentistry*.

[11] Lee J. J.-W., Kwon J.-Y., Chai H., Lucas P. W., Thompson V. P., Lawn B. R. (2009). Fracture modes in human teeth. *Journal of Dental Research*.

[12] Zheng J., Zeng Y., Wen J., Zheng L., Zhou Z. (2016). Impact wear behavior of human tooth enamel under simulated chewing conditions. *Journal of the Mechanical Behavior of Biomedical Materials*.

[13] Chai H. (2014). On the mechanical properties of tooth enamel under spherical indentation. *Acta Biomaterialia*.

[14] Li H., Zhou Z. R. (2001). Wear behavior of human teeth in dry and artificial saliva conditions. *Wear*.

[15] Lawn B. R., Lee J. J.-W. (2009). Analysis of fracture and deformation modes in teeth subjected to occlusal loading. *Acta Biomaterialia*.

[16] Levrini L., Di Benedetto G., Raspanti M. (2014). Dental wear: a scanning electron microscope study. *BioMed Research International*.

[17] Low I. M., Duraman N., Mahmood U. (2008). Mapping the structure, composition and mechanical properties of human teeth. *Materials Science and Engineering C: Materials for Biological Applications*.

[18] Park S., Wang D. H., Zhang D., Romberg E., Arola D. (2008). Mechanical properties of human enamel as a function of age and location in the tooth. *Journal of Materials Science: Materials in Medicine*.

[19] Xie Z., Swain M., Munroe P., Hoffman M. (2008). On the critical parameters that regulate the deformation behaviour of tooth enamel. *Biomaterials*.

[20] He L. H., Swain M. V. (2008). Understanding the mechanical behaviour of human enamel from its structural and compositional characteristics. *Journal of the Mechanical Behavior of Biomedical Materials*.

[21] Yahyazadehfar M., Bajaj D., Arola D. D. (2013). Hidden contributions of the enamel rods on the fracture resistance of human teeth. *Acta Biomaterialia*.

[22] Rivera C., Arola D., Ossa A. (2013). Indentation damage and crack repair in human enamel. *Journal of the Mechanical Behavior of Biomedical Materials*.

[23] Yahyazadehfar M., Arola D. (2015). The role of organic proteins on the crack growth resistance of human enamel. *Acta Biomaterialia*.

[24] Yahyazadehfar M., Zhang D., Arola D. (2016). On the importance of aging to the crack growth resistance of human enamel. *Acta Biomaterialia*.

[25] Wöltgens J. H. M., Bervoets T. J. M., Witjes F., Driessens F. C. M. (1981). Changes in the composition of the enamel of human premolar teeth shortly after eruption. *Archives of Oral Biolog*.

[26] Wöltgens J. H. M., Bervoets T. J. M., Witjes F., Driessens F. C. M. (1981). Effect of post-eruptive age on Ca and P loss from human enamel during demineralization in vitro. *Archives of Oral Biolog*.

[27] Oliveira M. A. H. D. M., Torres C. P., Gomes-Silva J. M. (2010). Microstructure and mineral composition of dental enamel of permanent and deciduous teeth. *Microscopy Research and Technique*.

[28] Yahyazadehfar M., Ivancik J., Majd H., An B., Zhang D., Arola D. (2014). On the mechanics of fatigue and fracture in teeth. *Applied Mechanics Reviews*.

[29] O'Brien S., Shaw J., Zhao X. (2014). Size dependent elastic modulus and mechanical resilience of dental enamel. *Journal of Biomechanics*.

[30] Habelitz S., Marshall S. J., Marshall G. W., Balooch M. (2001). Mechanical properties of human dental enamel on the nanometre scale. *Archives of Oral Biolog*.

