# Supplementary figures and images for: Enamel Microcracks Induced by Simulated Occlusal Wear in Mature, Immature, and Deciduous Teeth
Source: Biomed Res Int. 2018 Apr 16;2018:5658393. doi: 10.1155/2018/5658393 (PMC5926526; doi:10.1155/2018/5658393)

**
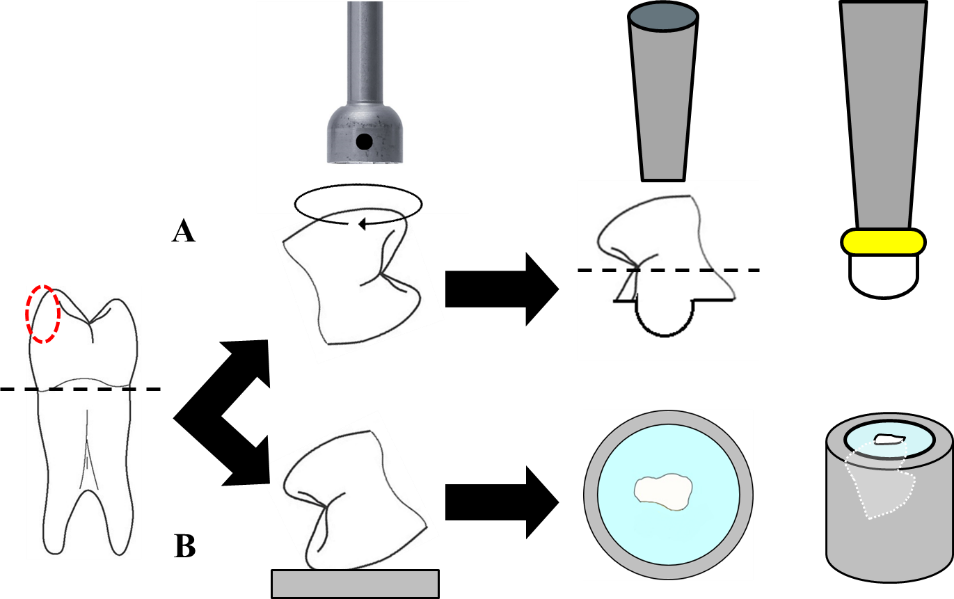
Supplementary Figure 1:**

**C:**


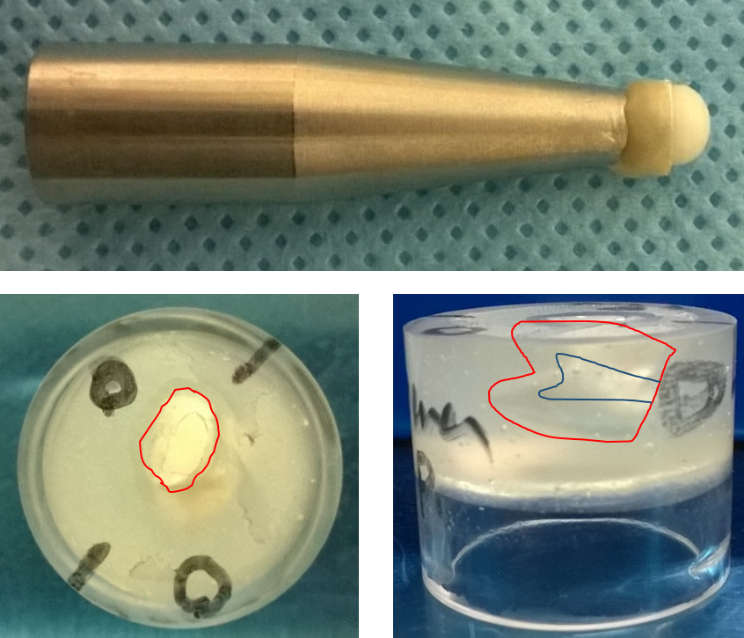

Supplement: Supplementary Figure 1 — A schematic representation of the preparation of the upper and lower specimens. A: the upper specimens were prepared by grinding the bucco-occlusal surfaces of the cusps and cutting the formed enamel hemispheres; they were then cemented on metallic styli. B: the lower specimens (study groups) were prepared by embedding crowns in acrylic rings and then molded in acrylic resin. C: actual images of the upper and lower specimens after polishing. Footnote of Supplementary Figure 1. The dotted line indicates the cutting direction; the red line indicates the final position of the crown. [file 5658393.f1.docx]

**
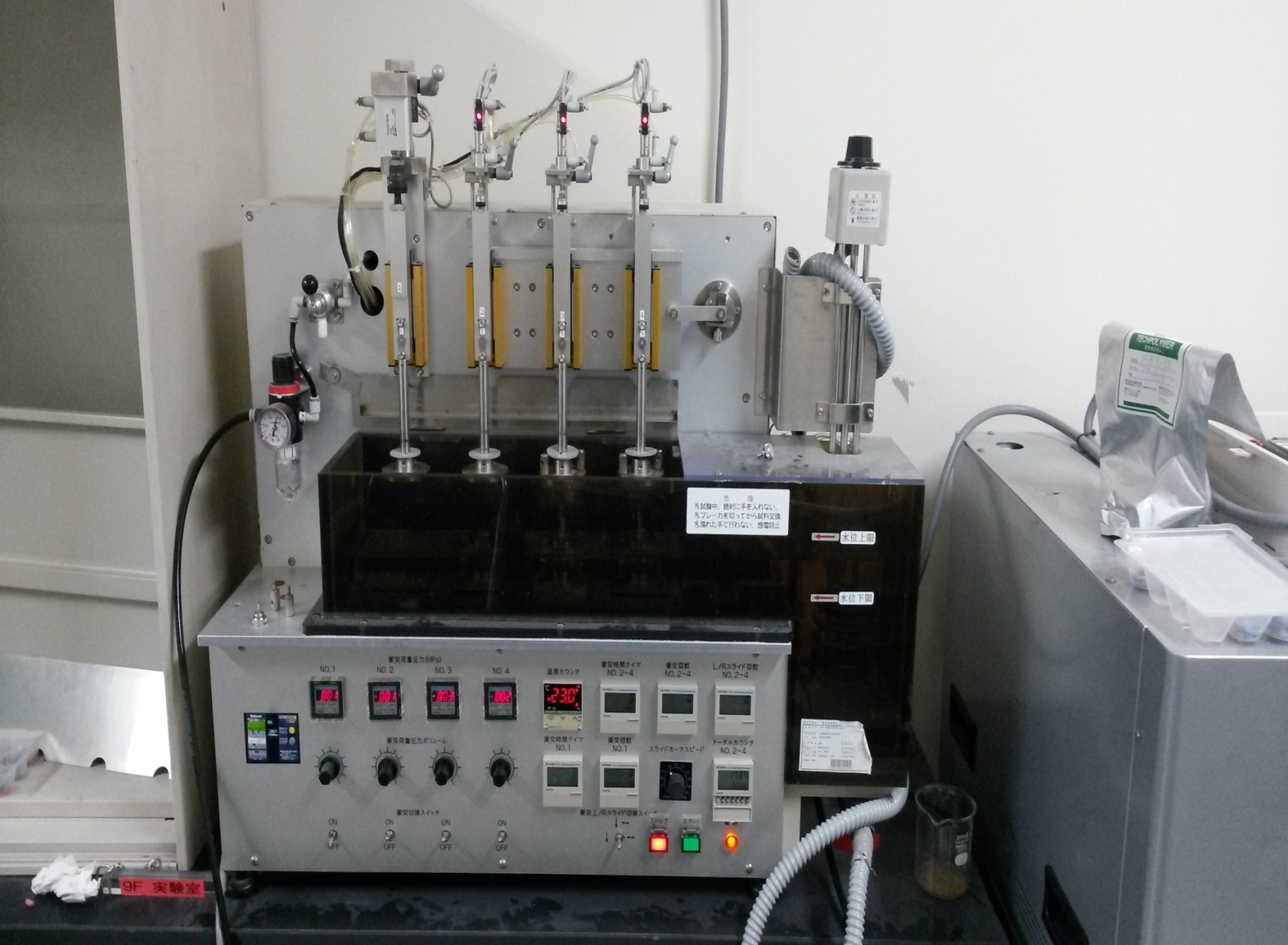

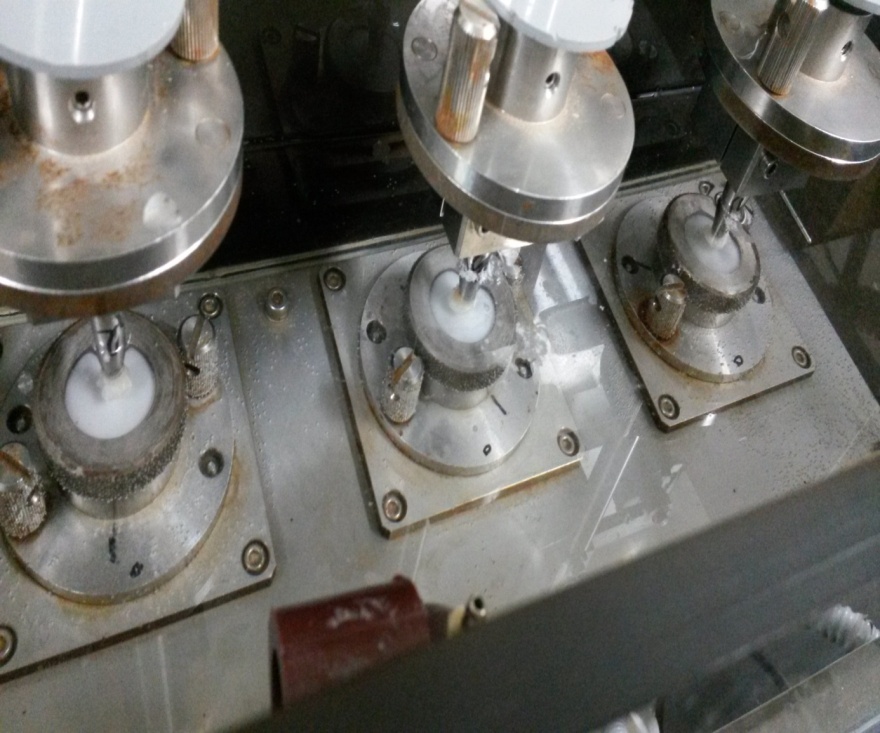
Supplementary Figure 2:**


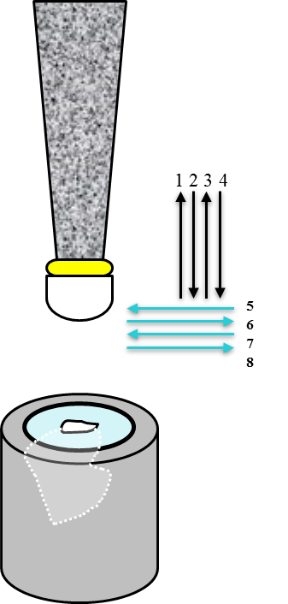

Supplement: Supplementary Figure 2 — Impact sliding wear testing (ISWT). Positions of the upper and lower enamel specimens below the water level in an impact sliding wear machine chamber. The whitish slurry of PMMA powder was placed in between specimens. The cyclic impact and sliding movements of the upper specimen against the lower specimen in relation to the initial contact position are indicated by numbered arrows (each arrow represents 1 mm). [file 5658393.f2.docx]

**Supplementary Figure 3:**


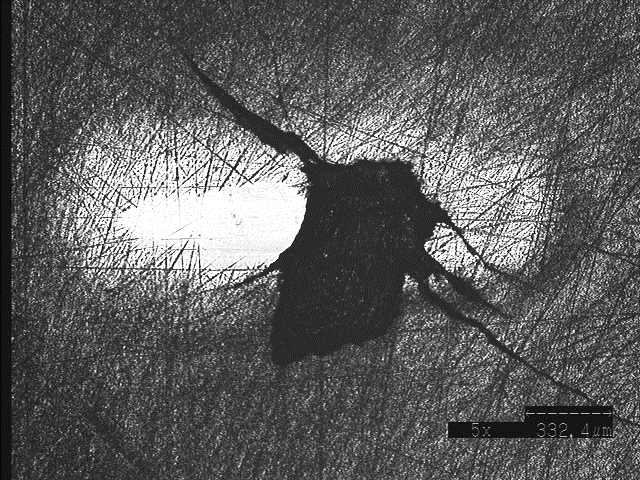

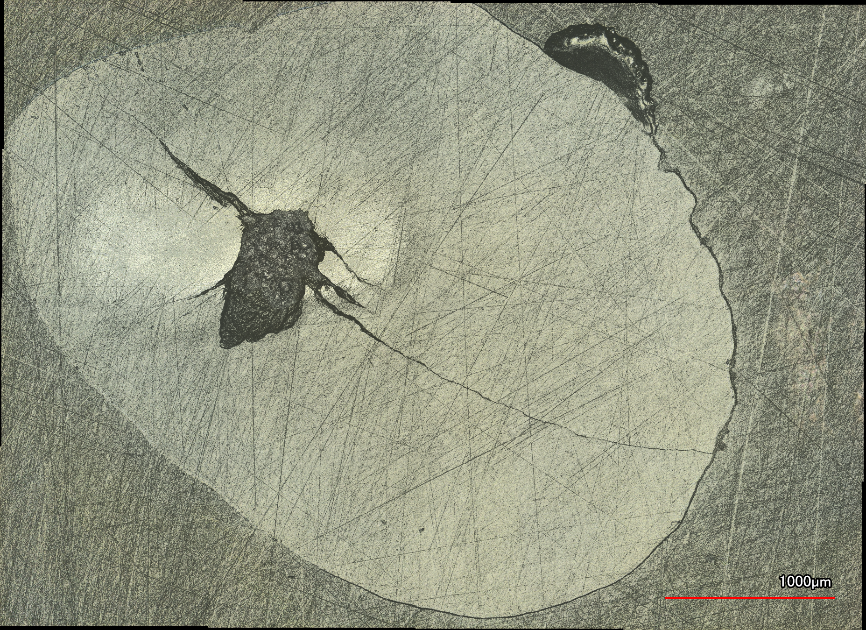

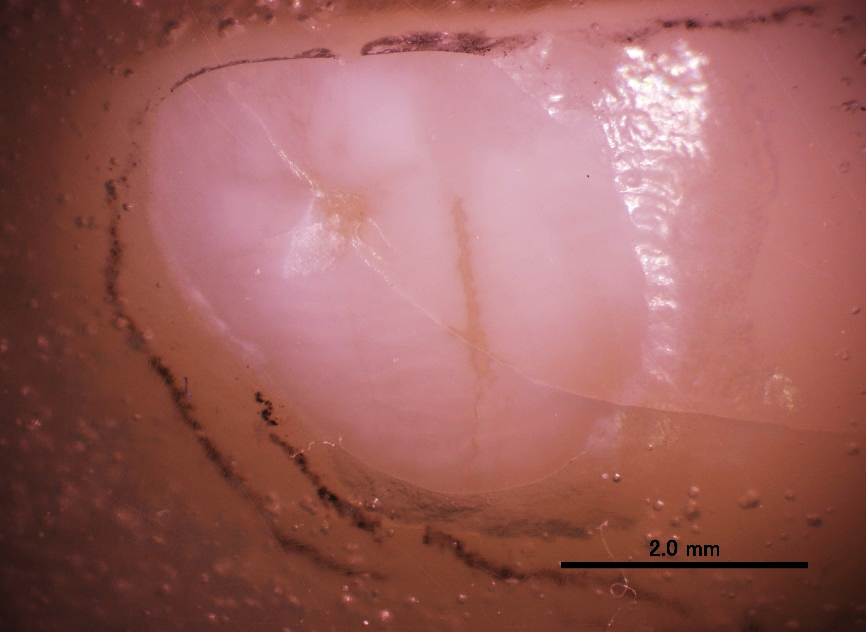

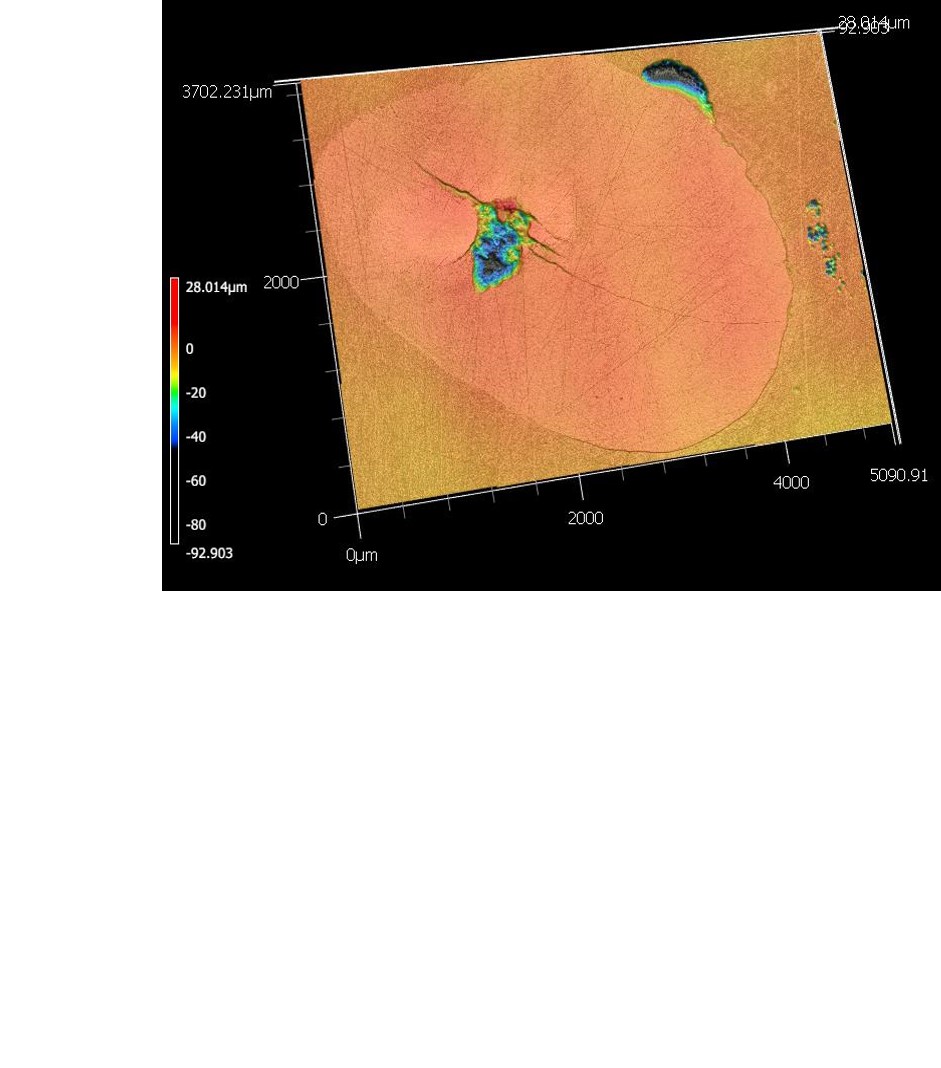


**B**

**A**

**C**

**D**

Supplement: Supplementary Figure 3 — Images of the enamel window of a deciduous enamel specimen under different microscopes. A: a stereomicroscope view. B: a scanning laser microscope and profilometer. C: a scanning confocal laser microscope. D: three-dimensional images were constructed with a depth scale (note the different scale measurements of each microscopic image). [file 5658393.f3.docx]
